# (1R*,2R*)-1-(7-Bromo-3-methoxy­naphthalen-2-yl)-4-(dimethyl­amino)-2-(naphthalen-1-yl)-1-phenyl­butan-2-ol

**DOI:** 10.1107/S1600536810005209

**Published:** 2010-02-13

**Authors:** Ping Liu, Wu Zhong, Pengfei Chen, Xiaohong Yang, Song Li

**Affiliations:** a Department of Medicinal Chemistry, School of Pharmacy, Jilin University, Changchun, 130021, People’s Republic of China; b Beijing Institute of Pharmacology and Toxicology, Beijing, 100850, People’s Republic of China

## Abstract

In the crystal structure of the title compound, C_33_H_32_BrNO_2_, the naphthalene ring system and the benzene ring are oriented at dihedral angles of 82.24 (4) and 79.53 (4)°, respectively, to the quinoline ring system. An intra­molecular O—H⋯N hydrogen bond occurs between the hydr­oxy H atom and the amine N atom.

## Related literature

For general background and the synthesis of diaryl­quinoline anti-tuberculosis drugs, see: Cohen (2004 ▶), Andries *et al.* (2005 ▶); Guillemont *et al.* (2004 ▶)
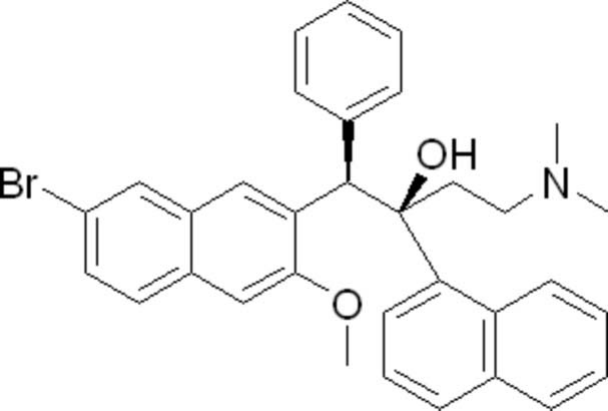


## Experimental

### 

#### Crystal data

C_33_H_32_BrNO_2_
*M* *_r_* = 554.51
Monoclinic, 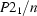

*a* = 12.716 (3) Å
*b* = 12.505 (4) Å
*c* = 17.771 (4) Å
β = 110.863 (7)°
*V* = 2640.6 (12) Å^3^
*Z* = 4
Mo *K*α radiation
μ = 1.59 mm^−1^
*T* = 113 K
0.22 × 0.20 × 0.16 mm

#### Data collection

Rigaku Saturn CCD area-detector diffractometer
Absorption correction: multi-scan (*CrystalClear*; Rigaku/MSC, 2005 ▶) *T*_min_ = 0.721, *T*_max_ = 0.785
21986 measured reflections
6297 independent reflections
4775 reflections with *I* > 2σ(*I*)
*R*_int_ = 0.039

#### Refinement

*R*[*F* ^2^ > 2σ(*F* ^2^)] = 0.030
*wR*(*F* ^2^) = 0.066
*S* = 1.01
6297 reflections
338 parameters
H-atom parameters constrained
Δρ_max_ = 0.44 e Å^−3^
Δρ_min_ = −0.36 e Å^−3^

### 

Data collection: *CrystalClear* (Rigaku/MSC, 2005 ▶); cell refinement: *CrystalClear*; data reduction: *CrystalClear*; program(s) used to solve structure: *SHELXS97* (Sheldrick, 2008 ▶); program(s) used to refine structure: *SHELXL97* (Sheldrick, 2008 ▶); molecular graphics: *SHELXTL* (Sheldrick, 2008 ▶); software used to prepare material for publication: *XCIF* in *SHELXTL*.

## Supplementary Material

Crystal structure: contains datablocks I, New_Global_Publ_Block. DOI: 10.1107/S1600536810005209/nc2176sup1.cif

Structure factors: contains datablocks I. DOI: 10.1107/S1600536810005209/nc2176Isup2.hkl

Additional supplementary materials:  crystallographic information; 3D view; checkCIF report

## Figures and Tables

**Table 1:** Hydrogen-bond geometry (Å, °)

*D*—H⋯*A*	*D*—H	H⋯*A*	*D*⋯*A*	*D*—H⋯*A*
O2—H2⋯N1	0.84	1.93	2.6988 (17)	151

**Fig. 1.**
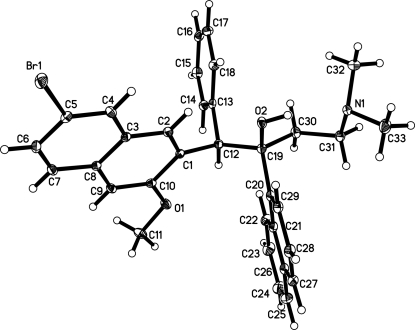
The molecular structure of title compound. Displacement ellipsoides a drawn at the 50% probability level. H atoms are presented as a small spheres of arbitrary radius.

## supplementary crystallographic information

### Comment

The compound (1*R**,2R*)-(1*R*,2S)-1-(6-bromo-2-methoxyquinolin-3-yl)-4- (dimethylamino)-2-(naphthalene-1-\ yl)-1-phenylbutan-2-ol, is a promising drug against tuberculosis (Andries *et al.*, 2005; Cohen, 2004 and Guillemont and Frans, 2004)). We modified this compound in order to get some more efficient antituberculosis drugs. To characterize our product its single crystal structure was determined.

In the molecule of the title compound (Fig. 1), the dihedral angle between the naphthalene ring (C20—C29) and the quinoline ring (C1—C10) amount to 82.244 (39)° whereas the benzene ring (C13—C18) is oriented with respect to the quinoline ring at a dihedral angle of 79.534 (39)°. In the structure an intramolecular O—H···N hydrogen bond is found (Tab. 1).

### Experimental

nBuLi (2.5M in hexanes, 4 ml, 10 mmol) was added slowly at 233 K under N_2 _to a solution of diisopropylamine (1.4 ml, 10 mmol) in THF (15 ml). The mixture was stirred at 233k for 30 min, then cooled to 195 K. Afterwards a solution of 3-benzyl-6-bromo-2-methoxynaphthalene (2.58 g, 9.2 mmol) in THF (20 ml) was added slowly. The mixture was stirred at 195 K for about 40 min  and then a solution of 3-(dimethylamino)-1-(naphthalen-1-yl)propan-1-one(2.9 g, 12.8 mmol) in THF (20 ml) was added slowly. The mixture was stirred at 195 K for 8 h, hydrolyzed with ice water at 233 K and extracted with ethyl acetate. The organic layer was separated, dried over MgSO_4_, filtered and the solvent was evaporated. The residue was purified by column chromatography over silica gel (eluent: petroleum ether/ethyl acetate, 50/1).Two fractions were collected (Guillemont *et al.*, 2004). On evaporation of the solvent (petroleum ether/ethyl acetate, 50/1) from fraction at room temperature in air single crystals of the title compound were obtained.

### Refinement

All H atoms were positioned with ideal geometry (O-H H atoms allowed to rotate but not to tip) and with d(C—H)= 0.93 Å for aromatic, 0.98 Å for CH, 0.97 Å for CH_2_ and 0.96 Å for CH_3_ atoms and were refined with *U*_iso_(H) = 1.2 *U*_eq_(C) for CH and CH_2_ H atoms and *U*_iso_(H) = 1.5 *U*_eq_(C) for CH_3_ and O-H H atoms.

### Crystal data

**Table:**

C_33_H_32_BrNO_2_	*F*(000) = 1152
*M**_r_* = 554.51	*D*_x_ = 1.395 Mg m^−^^3^
Monoclinic, *P*2_1_/*n*	Mo *K*α radiation, λ = 0.71073 Å
*a* = 12.716 (3) Å	Cell parameters from 9894 reflections
*b* = 12.505 (4) Å	θ = 1.7–27.9°
*c* = 17.771 (4) Å	µ = 1.59 mm^−^^1^
β = 110.863 (7)°	*T* = 113 K
*V* = 2640.6 (12) Å^3^	Prism, colorless
*Z* = 4	0.22 × 0.20 × 0.16 mm

### Data collection

**Table:**

Rigaku Saturn CCD area-detector diffractometer	6297 independent reflections
Radiation source: rotating anode	4775 reflections with *I* > 2σ(*I*)
multilayer	*R*_int_ = 0.039
Detector resolution: 14.63 pixels mm^-1^	θ_max_ = 27.9°, θ_min_ = 2.0°
ω and φ scans	*h* = −14→16
Absorption correction: multi-scan (*CrystalClear*; Rigaku/MSC, 2005)	*k* = −16→13
*T*_min_ = 0.721, *T*_max_ = 0.785	*l* = −23→23
21986 measured reflections	

### Refinement

**Table:**

Refinement on *F*^2^	Primary atom site location: structure-invariant direct methods
Least-squares matrix: full	Secondary atom site location: difference Fourier map
*R*[*F*^2^ > 2σ(*F*^2^)] = 0.030	Hydrogen site location: inferred from neighbouring sites
*wR*(*F*^2^) = 0.066	H-atom parameters constrained
*S* = 1.01	*w* = 1/[σ^2^(*F*_o_^2^) + (0.026*P*)^2^] where *P* = (*F*_o_^2^ + 2*F*_c_^2^)/3
6297 reflections	(Δ/σ)_max_ = 0.004
338 parameters	Δρ_max_ = 0.44 e Å^−^^3^
0 restraints	Δρ_min_ = −0.36 e Å^−^^3^

### Special details

**Table:**

Geometry. All e.s.d.'s (except the e.s.d. in the dihedral angle between two l.s. planes) are estimated using the full covariance matrix. The cell e.s.d.'s are taken into account individually in the estimation of e.s.d.'s in distances, angles and torsion angles; correlations between e.s.d.'s in cell parameters are only used when they are defined by crystal symmetry. An approximate (isotropic) treatment of cell e.s.d.'s is used for estimating e.s.d.'s involving l.s. planes.
Refinement. Refinement of *F*^2^ against ALL reflections. The weighted *R*-factor wR and goodness of fit *S* are based on *F*^2^, conventional *R*-factors *R* are based on *F*, with *F* set to zero for negative *F*^2^. The threshold expression of *F*^2^ > σ(*F*^2^) is used only for calculating *R*-factors(gt) etc. and is not relevant to the choice of reflections for refinement. *R*-factors based on *F*^2^ are statistically about twice as large as those based on *F*, and *R*- factors based on ALL data will be even larger.

### Fractional atomic coordinates and isotropic or equivalent isotropic displacement parameters (Å^2^)

**Table:**

	*x*	*y*	*z*	*U*_iso_*/*U*_eq_	
Br1	0.855107 (15)	−0.301941 (13)	0.033962 (11)	0.02661 (6)	
O1	1.09459 (9)	0.30832 (8)	0.16894 (6)	0.0200 (3)	
O2	0.70336 (9)	0.24821 (9)	0.13087 (6)	0.0179 (2)	
H2	0.6433	0.2667	0.1368	0.027*	
N1	0.54575 (10)	0.37440 (11)	0.15453 (7)	0.0183 (3)	
C1	0.92349 (13)	0.21144 (12)	0.11745 (8)	0.0149 (3)	
C2	0.86920 (13)	0.11576 (12)	0.09563 (8)	0.0168 (3)	
H2A	0.7898	0.1137	0.0814	0.020*	
C3	0.92691 (13)	0.01955 (12)	0.09339 (8)	0.0155 (3)	
C4	0.86996 (13)	−0.07937 (12)	0.06968 (9)	0.0180 (3)	
H4	0.7908	−0.0835	0.0566	0.022*	
C5	0.92952 (14)	−0.16813 (13)	0.06576 (9)	0.0187 (4)	
C6	1.04680 (14)	−0.16596 (13)	0.08397 (9)	0.0197 (4)	
H6	1.0862	−0.2291	0.0802	0.024*	
C7	1.10257 (13)	−0.07195 (13)	0.10705 (9)	0.0189 (4)	
H7	1.1816	−0.0699	0.1194	0.023*	
C8	1.04561 (13)	0.02292 (13)	0.11305 (8)	0.0154 (3)	
C9	1.10209 (13)	0.12096 (13)	0.13816 (8)	0.0167 (3)	
H9	1.1815	0.1241	0.1524	0.020*	
C10	1.04431 (13)	0.21130 (12)	0.14228 (9)	0.0161 (3)	
C11	1.21478 (13)	0.31054 (13)	0.19889 (10)	0.0248 (4)	
H11A	1.2438	0.2567	0.2413	0.037*	
H11B	1.2410	0.3815	0.2210	0.037*	
H11C	1.2420	0.2949	0.1549	0.037*	
C12	0.86391 (13)	0.31938 (12)	0.10741 (9)	0.0153 (3)	
H12	0.9250	0.3732	0.1311	0.018*	
C13	0.81297 (13)	0.34627 (13)	0.01755 (9)	0.0156 (3)	
C14	0.86628 (13)	0.42324 (12)	−0.01233 (9)	0.0185 (3)	
H14	0.9311	0.4582	0.0236	0.022*	
C15	0.82752 (14)	0.45026 (13)	−0.09287 (9)	0.0221 (4)	
H15	0.8658	0.5027	−0.1121	0.027*	
C16	0.73263 (14)	0.40062 (13)	−0.14546 (9)	0.0226 (4)	
H16	0.7051	0.4193	−0.2009	0.027*	
C17	0.67777 (14)	0.32337 (13)	−0.11695 (9)	0.0229 (4)	
H17	0.6125	0.2892	−0.1529	0.028*	
C18	0.71826 (14)	0.29622 (13)	−0.03610 (9)	0.0200 (4)	
H18	0.6809	0.2427	−0.0171	0.024*	
C19	0.78158 (13)	0.33436 (12)	0.15313 (9)	0.0152 (3)	
C20	0.84763 (13)	0.33288 (12)	0.24544 (9)	0.0165 (3)	
C21	0.93020 (13)	0.41240 (13)	0.28752 (9)	0.0185 (3)	
C22	0.96876 (14)	0.49707 (13)	0.25033 (10)	0.0226 (4)	
H22	0.9379	0.5042	0.1934	0.027*	
C23	1.04865 (15)	0.56833 (14)	0.29379 (10)	0.0298 (4)	
H23	1.0728	0.6229	0.2665	0.036*	
C24	1.09580 (15)	0.56233 (15)	0.37820 (10)	0.0330 (5)	
H24	1.1507	0.6128	0.4080	0.040*	
C25	1.06141 (15)	0.48290 (15)	0.41645 (10)	0.0290 (4)	
H25	1.0932	0.4784	0.4735	0.035*	
C26	0.97938 (14)	0.40671 (14)	0.37352 (9)	0.0213 (4)	
C27	0.94584 (14)	0.32554 (13)	0.41524 (9)	0.0231 (4)	
H27	0.9772	0.3231	0.4724	0.028*	
C28	0.86905 (14)	0.25075 (14)	0.37456 (9)	0.0231 (4)	
H28	0.8481	0.1956	0.4032	0.028*	
C29	0.82034 (14)	0.25491 (13)	0.28971 (9)	0.0197 (4)	
H29	0.7669	0.2019	0.2623	0.024*	
C30	0.71441 (13)	0.43988 (13)	0.12888 (9)	0.0192 (4)	
H30A	0.7680	0.5004	0.1402	0.023*	
H30B	0.6716	0.4389	0.0702	0.023*	
C31	0.63248 (13)	0.45846 (13)	0.17306 (9)	0.0200 (4)	
H31A	0.6748	0.4598	0.2318	0.024*	
H31B	0.5956	0.5289	0.1573	0.024*	
C32	0.45593 (14)	0.39331 (16)	0.07646 (9)	0.0297 (4)	
H32A	0.4016	0.3344	0.0646	0.045*	
H32B	0.4885	0.3974	0.0342	0.045*	
H32C	0.4177	0.4607	0.0784	0.045*	
C33	0.49847 (15)	0.36798 (15)	0.21797 (10)	0.0299 (4)	
H33A	0.4647	0.4369	0.2228	0.045*	
H33B	0.5582	0.3506	0.2692	0.045*	
H33C	0.4406	0.3121	0.2046	0.045*	

### Atomic displacement parameters (Å^2^)

**Table:**

	*U*^11^	*U*^22^	*U*^33^	*U*^12^	*U*^13^	*U*^23^
Br1	0.02826 (10)	0.01699 (10)	0.03531 (11)	−0.00165 (8)	0.01222 (8)	−0.00535 (8)
O1	0.0141 (6)	0.0170 (6)	0.0266 (6)	−0.0019 (5)	0.0043 (5)	−0.0017 (5)
O2	0.0150 (6)	0.0170 (6)	0.0236 (6)	−0.0014 (5)	0.0094 (5)	−0.0025 (5)
N1	0.0148 (7)	0.0228 (8)	0.0176 (6)	0.0014 (6)	0.0061 (5)	−0.0020 (6)
C1	0.0155 (8)	0.0185 (9)	0.0117 (7)	0.0017 (7)	0.0060 (6)	0.0009 (6)
C2	0.0150 (8)	0.0199 (9)	0.0157 (7)	−0.0003 (7)	0.0057 (6)	−0.0008 (7)
C3	0.0185 (8)	0.0170 (9)	0.0116 (7)	0.0018 (7)	0.0060 (6)	0.0012 (7)
C4	0.0162 (8)	0.0166 (9)	0.0206 (8)	0.0018 (7)	0.0058 (6)	0.0029 (7)
C5	0.0239 (9)	0.0169 (9)	0.0158 (8)	−0.0010 (7)	0.0076 (7)	−0.0016 (7)
C6	0.0244 (9)	0.0187 (9)	0.0182 (8)	0.0065 (7)	0.0104 (7)	0.0020 (7)
C7	0.0154 (8)	0.0238 (9)	0.0183 (8)	0.0043 (7)	0.0070 (6)	0.0031 (7)
C8	0.0187 (8)	0.0179 (9)	0.0102 (7)	0.0029 (7)	0.0059 (6)	0.0027 (7)
C9	0.0120 (8)	0.0212 (9)	0.0169 (7)	0.0003 (7)	0.0052 (6)	0.0023 (7)
C10	0.0166 (8)	0.0186 (9)	0.0129 (7)	−0.0021 (7)	0.0051 (6)	0.0008 (7)
C11	0.0157 (8)	0.0244 (10)	0.0292 (9)	−0.0039 (8)	0.0018 (7)	0.0036 (8)
C12	0.0142 (8)	0.0151 (9)	0.0163 (7)	−0.0017 (7)	0.0051 (6)	−0.0014 (7)
C13	0.0174 (8)	0.0137 (8)	0.0179 (8)	0.0033 (7)	0.0089 (6)	−0.0012 (7)
C14	0.0198 (9)	0.0149 (8)	0.0209 (8)	−0.0005 (7)	0.0074 (7)	−0.0013 (7)
C15	0.0267 (9)	0.0176 (9)	0.0244 (9)	0.0026 (8)	0.0120 (7)	0.0057 (7)
C16	0.0310 (10)	0.0214 (9)	0.0159 (8)	0.0078 (8)	0.0091 (7)	0.0023 (7)
C17	0.0226 (9)	0.0234 (10)	0.0192 (8)	0.0010 (7)	0.0031 (7)	−0.0030 (7)
C18	0.0205 (9)	0.0192 (9)	0.0213 (8)	−0.0021 (7)	0.0085 (7)	−0.0009 (7)
C19	0.0158 (8)	0.0127 (8)	0.0178 (7)	−0.0016 (7)	0.0067 (6)	−0.0012 (7)
C20	0.0172 (8)	0.0155 (8)	0.0189 (8)	0.0036 (7)	0.0090 (7)	−0.0008 (7)
C21	0.0188 (9)	0.0185 (9)	0.0197 (8)	0.0021 (7)	0.0087 (7)	−0.0028 (7)
C22	0.0264 (9)	0.0194 (9)	0.0233 (8)	−0.0022 (8)	0.0103 (7)	−0.0045 (8)
C23	0.0333 (11)	0.0245 (10)	0.0347 (10)	−0.0084 (9)	0.0158 (8)	−0.0072 (9)
C24	0.0290 (10)	0.0348 (12)	0.0334 (10)	−0.0101 (9)	0.0088 (8)	−0.0138 (9)
C25	0.0246 (10)	0.0361 (11)	0.0233 (9)	−0.0007 (9)	0.0047 (7)	−0.0083 (8)
C26	0.0189 (9)	0.0233 (9)	0.0222 (8)	0.0039 (8)	0.0079 (7)	−0.0046 (7)
C27	0.0243 (9)	0.0281 (10)	0.0171 (8)	0.0060 (8)	0.0075 (7)	−0.0008 (7)
C28	0.0290 (10)	0.0224 (10)	0.0219 (8)	0.0057 (8)	0.0139 (7)	0.0062 (8)
C29	0.0212 (9)	0.0169 (9)	0.0225 (8)	0.0019 (8)	0.0095 (7)	−0.0002 (7)
C30	0.0198 (9)	0.0168 (9)	0.0227 (8)	0.0016 (7)	0.0098 (7)	0.0026 (7)
C31	0.0213 (9)	0.0160 (9)	0.0225 (8)	0.0032 (7)	0.0077 (7)	−0.0014 (7)
C32	0.0234 (10)	0.0404 (12)	0.0210 (8)	0.0028 (9)	0.0026 (7)	−0.0052 (8)
C33	0.0299 (10)	0.0354 (11)	0.0307 (10)	−0.0008 (9)	0.0185 (8)	−0.0008 (9)

### Geometric parameters (Å, °)

**Table:**

Br1—C5	1.9063 (16)	C16—C17	1.389 (2)
O1—C10	1.3746 (18)	C16—H16	0.9500
O1—C11	1.4285 (19)	C17—C18	1.385 (2)
O2—C19	1.4235 (18)	C17—H17	0.9500
O2—H2	0.8400	C18—H18	0.9500
N1—C33	1.457 (2)	C19—C30	1.547 (2)
N1—C32	1.4686 (19)	C19—C20	1.554 (2)
N1—C31	1.473 (2)	C20—C29	1.373 (2)
C1—C2	1.367 (2)	C20—C21	1.446 (2)
C1—C10	1.440 (2)	C21—C22	1.424 (2)
C1—C12	1.527 (2)	C21—C26	1.432 (2)
C2—C3	1.417 (2)	C22—C23	1.365 (2)
C2—H2A	0.9500	C22—H22	0.9500
C3—C4	1.420 (2)	C23—C24	1.405 (2)
C3—C8	1.424 (2)	C23—H23	0.9500
C4—C5	1.359 (2)	C24—C25	1.361 (2)
C4—H4	0.9500	C24—H24	0.9500
C5—C6	1.409 (2)	C25—C26	1.418 (2)
C6—C7	1.359 (2)	C25—H25	0.9500
C6—H6	0.9500	C26—C27	1.410 (2)
C7—C8	1.414 (2)	C27—C28	1.359 (2)
C7—H7	0.9500	C27—H27	0.9500
C8—C9	1.411 (2)	C28—C29	1.412 (2)
C9—C10	1.364 (2)	C28—H28	0.9500
C9—H9	0.9500	C29—H29	0.9500
C11—H11A	0.9800	C30—C31	1.529 (2)
C11—H11B	0.9800	C30—H30A	0.9900
C11—H11C	0.9800	C30—H30B	0.9900
C12—C13	1.532 (2)	C31—H31A	0.9900
C12—C19	1.548 (2)	C31—H31B	0.9900
C12—H12	1.0000	C32—H32A	0.9800
C13—C14	1.387 (2)	C32—H32B	0.9800
C13—C18	1.390 (2)	C32—H32C	0.9800
C14—C15	1.380 (2)	C33—H33A	0.9800
C14—H14	0.9500	C33—H33B	0.9800
C15—C16	1.383 (2)	C33—H33C	0.9800
C15—H15	0.9500		
			
C10—O1—C11	116.76 (12)	C17—C18—H18	119.6
C19—O2—H2	109.5	C13—C18—H18	119.6
C33—N1—C32	110.09 (13)	O2—C19—C30	107.83 (12)
C33—N1—C31	110.41 (12)	O2—C19—C12	107.39 (12)
C32—N1—C31	111.38 (13)	C30—C19—C12	111.50 (12)
C2—C1—C10	117.52 (14)	O2—C19—C20	110.23 (12)
C2—C1—C12	124.14 (14)	C30—C19—C20	109.96 (12)
C10—C1—C12	117.89 (14)	C12—C19—C20	109.89 (12)
C1—C2—C3	122.47 (15)	C29—C20—C21	118.51 (14)
C1—C2—H2A	118.8	C29—C20—C19	117.48 (14)
C3—C2—H2A	118.8	C21—C20—C19	123.89 (13)
C2—C3—C4	122.25 (14)	C22—C21—C26	116.13 (14)
C2—C3—C8	118.84 (14)	C22—C21—C20	125.29 (14)
C4—C3—C8	118.87 (14)	C26—C21—C20	118.58 (14)
C5—C4—C3	119.48 (15)	C23—C22—C21	122.18 (15)
C5—C4—H4	120.3	C23—C22—H22	118.9
C3—C4—H4	120.3	C21—C22—H22	118.9
C4—C5—C6	122.41 (15)	C22—C23—C24	121.17 (17)
C4—C5—Br1	120.34 (13)	C22—C23—H23	119.4
C6—C5—Br1	117.25 (12)	C24—C23—H23	119.4
C7—C6—C5	118.81 (15)	C25—C24—C23	118.78 (17)
C7—C6—H6	120.6	C25—C24—H24	120.6
C5—C6—H6	120.6	C23—C24—H24	120.6
C6—C7—C8	121.50 (15)	C24—C25—C26	121.83 (16)
C6—C7—H7	119.3	C24—C25—H25	119.1
C8—C7—H7	119.3	C26—C25—H25	119.1
C9—C8—C7	122.44 (14)	C27—C26—C25	120.21 (15)
C9—C8—C3	118.64 (14)	C27—C26—C21	119.88 (15)
C7—C8—C3	118.93 (15)	C25—C26—C21	119.91 (15)
C10—C9—C8	120.90 (15)	C28—C27—C26	120.68 (15)
C10—C9—H9	119.5	C28—C27—H27	119.7
C8—C9—H9	119.5	C26—C27—H27	119.7
C9—C10—O1	123.80 (14)	C27—C28—C29	120.08 (15)
C9—C10—C1	121.41 (15)	C27—C28—H28	120.0
O1—C10—C1	114.79 (13)	C29—C28—H28	120.0
O1—C11—H11A	109.5	C20—C29—C28	122.26 (15)
O1—C11—H11B	109.5	C20—C29—H29	118.9
H11A—C11—H11B	109.5	C28—C29—H29	118.9
O1—C11—H11C	109.5	C31—C30—C19	113.34 (13)
H11A—C11—H11C	109.5	C31—C30—H30A	108.9
H11B—C11—H11C	109.5	C19—C30—H30A	108.9
C1—C12—C13	108.90 (12)	C31—C30—H30B	108.9
C1—C12—C19	116.47 (12)	C19—C30—H30B	108.9
C13—C12—C19	113.99 (13)	H30A—C30—H30B	107.7
C1—C12—H12	105.5	N1—C31—C30	111.68 (13)
C13—C12—H12	105.5	N1—C31—H31A	109.3
C19—C12—H12	105.5	C30—C31—H31A	109.3
C14—C13—C18	118.16 (14)	N1—C31—H31B	109.3
C14—C13—C12	117.94 (14)	C30—C31—H31B	109.3
C18—C13—C12	123.88 (14)	H31A—C31—H31B	107.9
C15—C14—C13	121.63 (15)	N1—C32—H32A	109.5
C15—C14—H14	119.2	N1—C32—H32B	109.5
C13—C14—H14	119.2	H32A—C32—H32B	109.5
C14—C15—C16	119.64 (15)	N1—C32—H32C	109.5
C14—C15—H15	120.2	H32A—C32—H32C	109.5
C16—C15—H15	120.2	H32B—C32—H32C	109.5
C15—C16—C17	119.78 (15)	N1—C33—H33A	109.5
C15—C16—H16	120.1	N1—C33—H33B	109.5
C17—C16—H16	120.1	H33A—C33—H33B	109.5
C18—C17—C16	119.97 (15)	N1—C33—H33C	109.5
C18—C17—H17	120.0	H33A—C33—H33C	109.5
C16—C17—H17	120.0	H33B—C33—H33C	109.5
C17—C18—C13	120.81 (15)		
			
C10—C1—C2—C3	−2.8 (2)	C14—C13—C18—C17	−0.6 (2)
C12—C1—C2—C3	169.34 (13)	C12—C13—C18—C17	−178.95 (15)
C1—C2—C3—C4	−179.16 (14)	C1—C12—C19—O2	−54.08 (16)
C1—C2—C3—C8	−1.3 (2)	C13—C12—C19—O2	74.07 (16)
C2—C3—C4—C5	177.46 (14)	C1—C12—C19—C30	−171.99 (12)
C8—C3—C4—C5	−0.4 (2)	C13—C12—C19—C30	−43.84 (17)
C3—C4—C5—C6	−0.4 (2)	C1—C12—C19—C20	65.83 (17)
C3—C4—C5—Br1	−179.92 (10)	C13—C12—C19—C20	−166.02 (12)
C4—C5—C6—C7	0.7 (2)	O2—C19—C20—C29	−1.59 (19)
Br1—C5—C6—C7	−179.83 (11)	C30—C19—C20—C29	117.16 (15)
C5—C6—C7—C8	0.0 (2)	C12—C19—C20—C29	−119.75 (15)
C6—C7—C8—C9	178.93 (14)	O2—C19—C20—C21	−177.43 (13)
C6—C7—C8—C3	−0.8 (2)	C30—C19—C20—C21	−58.69 (18)
C2—C3—C8—C9	3.3 (2)	C12—C19—C20—C21	64.40 (18)
C4—C3—C8—C9	−178.72 (13)	C29—C20—C21—C22	178.94 (15)
C2—C3—C8—C7	−176.94 (13)	C19—C20—C21—C22	−5.3 (2)
C4—C3—C8—C7	1.0 (2)	C29—C20—C21—C26	−0.4 (2)
C7—C8—C9—C10	179.15 (14)	C19—C20—C21—C26	175.44 (14)
C3—C8—C9—C10	−1.1 (2)	C26—C21—C22—C23	0.6 (2)
C8—C9—C10—O1	177.60 (13)	C20—C21—C22—C23	−178.76 (16)
C8—C9—C10—C1	−3.2 (2)	C21—C22—C23—C24	−1.0 (3)
C11—O1—C10—C9	−4.5 (2)	C22—C23—C24—C25	0.8 (3)
C11—O1—C10—C1	176.23 (13)	C23—C24—C25—C26	−0.2 (3)
C2—C1—C10—C9	5.1 (2)	C24—C25—C26—C27	−179.99 (17)
C12—C1—C10—C9	−167.56 (13)	C24—C25—C26—C21	−0.2 (3)
C2—C1—C10—O1	−175.57 (12)	C22—C21—C26—C27	179.84 (15)
C12—C1—C10—O1	11.76 (19)	C20—C21—C26—C27	−0.8 (2)
C2—C1—C12—C13	−69.92 (18)	C22—C21—C26—C25	0.0 (2)
C10—C1—C12—C13	102.23 (15)	C20—C21—C26—C25	179.38 (15)
C2—C1—C12—C19	60.67 (19)	C25—C26—C27—C28	−178.59 (16)
C10—C1—C12—C19	−127.18 (15)	C21—C26—C27—C28	1.6 (2)
C1—C12—C13—C14	−105.02 (16)	C26—C27—C28—C29	−1.2 (2)
C19—C12—C13—C14	123.06 (15)	C21—C20—C29—C28	0.8 (2)
C1—C12—C13—C18	73.29 (18)	C19—C20—C29—C28	−175.28 (14)
C19—C12—C13—C18	−58.6 (2)	C27—C28—C29—C20	0.0 (2)
C18—C13—C14—C15	0.0 (2)	O2—C19—C30—C31	62.77 (16)
C12—C13—C14—C15	178.37 (14)	C12—C19—C30—C31	−179.59 (13)
C13—C14—C15—C16	0.6 (2)	C20—C19—C30—C31	−57.44 (17)
C14—C15—C16—C17	−0.6 (2)	C33—N1—C31—C30	157.51 (13)
C15—C16—C17—C18	−0.1 (2)	C32—N1—C31—C30	−79.86 (16)
C16—C17—C18—C13	0.7 (2)	C19—C30—C31—N1	−62.24 (17)

### Hydrogen-bond geometry (Å, °)

**Table:**

*D*—H···*A*	*D*—H	H···*A*	*D*···*A*	*D*—H···*A*
O2—H2···N1	0.84	1.93	2.6988 (17)	151
